# Effect of Clinker Binder and Aggregates on Autogenous Healing in Post-Crack Flexural Behavior of Concrete Members

**DOI:** 10.3390/ma13204516

**Published:** 2020-10-12

**Authors:** Kwang-Myong Lee, Young-Cheol Choi, Byoungsun Park, Jinkyo F. Choo, Sung-Won Yoo

**Affiliations:** 1 Department of Civil and Environmental System Engineering, Sungkyunkwan University, 2066 Seobu-ro, Jangan-gu, Suwon-si 16419, Gyeonggi-do, Korea; leekm79@skku.edu; 2 Department of Civil and Environmental Engineering, Gachon University, 1342 Seongnamdae-ro, Sujeong-gu, Seongnam-si 13120, Gyeonggi-do, Korea; zerofe@gachon.ac.kr; 3 Construction Technology Research Center, Korea Conformity Laboratories, 199 Gasan digital 1-ro, Geumcheon-gu, Seoul 08503, Korea; pbs0927@kcl.re.kr; 4 Department of Energy Engineering, Konkuk University, 120 Neungdong-ro, Gwangjin-gu, Seoul 05029, Korea

**Keywords:** crack healing, clinker binder and aggregate, flexural performance, water flow test, curing

## Abstract

Crack healing has been studied extensively to protect reinforced concrete structures from the ingress of harmful ions. Research examining the regain in the mechanical properties of self-healing composites has focused mostly on the computation of the healing ratio based on the measurement of the tensile and compressive strengths but with poor regard for the flexural performance. However, the regain in the flexural performance should also be investigated for design purposes. The present study performs flexural testing on reinforced concrete members using crushed clinker binder and aggregates as well as crystalline admixtures as healing agents. Healing ratios of 100% for crack widths smaller than 200 μm and 85% to 90% for crack widths of 250 μm were observed according to the admixing of clinker binder and aggregates. Water flow test showed that the members replacing binder by 100% of clinker achieved the best crack healing performance. The crack healing property of concrete improved to some extent the rebar yield load, the members’ ultimate load and energy absorption capacity and ductility index. The crack distribution density from the observed crack patterns confirmed the crack healing effect provided by clinker powder. The fine grain size of clinker made it possible to replace fine aggregates and longer healing time increased the crack healing effect.

## 1. Introduction

Reinforced concrete can deteriorate due to a variety of reasons among which the corrosion of reinforcing steel is a leading cause. Reinforced steel corrodes due to the ingress of harmful ions like chlorides and sulfates. Once steel starts to corrode, the resulting rust occupies a larger volume, which creates tensile stresses in concrete and eventually leads to cracking, delamination and spalling of concrete. In turn, such cracking of concrete accelerates the penetration of harmful ions that worsens the degradation of the reinforced concrete structure [1]. Implementing repair of concrete cracking in due time is thus fundamental in extending the lifespan of the structure. However, apart from being costly and labor-intensive, manual repair is often inaccessible in offshore and underground concrete structures, which are likely to experience aggravated degradation and a shortened lifespan.

The restless search for durability and resilience of concrete structures has led some researchers to focus on self-healing concrete composites with the built-in ability of repairing narrow cracks without human or external intervention [2,3,4,5]. Two major types of self-healing concretes have emerged: the autogenous type [4], which is achieved by autogenous healing materials such as mineral admixtures like ground-granulated blast-furnace slag (GGBFS), silica fume or fly ash, fibers and nanofillers [6,7,8,9,10,11,12]; and, the autonomous type [3], which is realized by unconventional engineered additions such as shape memory alloys, capsules, polymers or bacteria to seal the cracks [13,14,15,16,17,18,19,20,21,22,23,24]. Autogenous healing is an old and well-known phenomenon that originates naturally from the cementitious material like the hydration of clinker minerals or the carbonation of calcium hydroxide while autonomous healing requires a trigger to activate the process. Both types have been proven to restore the mechanical properties and durability of the concrete structure to some extent but are believed to be only capable of repairing cracks within a few hundreds of micrometers, meaning structural damage cannot be repaired. To date, the autonomous healing method has shown better performance in healing cracks than most of the autogenous healing methods which have been seen to heal cracks with widths narrower than 150 μm [5].

Most studies assessing the self-healing performance of concrete have focused on the recovery of durability through the evaluation of the crack filling ratio and the reduction of penetration [25,26]. The methods usually adopted are chloride permeability test, water permeability test, isothermal calorimetry, crack closing test, etc. [12,25]. However, as pointed out by Guo and Chidiac [27], self-healing of concrete is made up of two concurrent concepts: self-sealing of cracks, which requires plugging of openings for durability, and self-healing of cracks, which refers to recovery of mechanical properties for strength. Therefore, some studies also conducted tensile and compressive strength tests, flexural and ultrasonic pulse velocity tests, etc. to assess the recovery of mechanical properties. When it comes to flexure, studies on self-healing concrete focus mainly on crack repair rather than mechanical performance. It is also noteworthy that the regain in mechanical properties may become meaningless if the repaired crack is weaker than the concrete matrix, subsequent cracks may develop at the same location when the healing agent is exhausted [2,27].

Considering the lifespan of the concrete structure and to be free of the exhaustion of the healing agent, this study pays attention to the autogenous healing. A recent study showed that the autogenous healing could be enhanced using GGBFS and crystalline admixture rather than using fly ash [28]. The results of this work confirmed that concretes incorporating supplementary cementitious materials like GGBFS can develop superior self-healing properties owing to the significant amount of unhydrated particles present in its microstructures as well as improved mechanical and permeation properties when stressed by mechanical loads [29]. On the other hand, another study investigated the proper distribution of cement particle sizes providing a suitable amount of Ca(OH)_2_ and unhydrated cement to improve the self-healing ability of concrete [30]. Moreover, Berger [31] and Allahverdi [32] reported that the use of cement clinker aggregates significantly improves the concrete properties like the compressive strength, chloride penetration depth and water absorption. Besides, the phases present in the cement clinker binder are known to yield a strong solid with low porosity and offer protection to chloride ingress.

Accordingly, this study examines the autogenous healing of concrete containing clinker binder and aggregates as well as crystalline admixtures as healing agents. Crack healing effect is investigated through crack closing and water flow tests. In addition, even if the flexural behavior depends to a large extent on the steel reinforcement, bending tests are conducted on full-scale test members to investigate any eventual recovery in the flexural mechanical performance brought by the autogenous healing. For the tests, three different concrete mixes and four different loading and curing conditions were chosen as test variables. Cracking was induced by pre-loading the specimens at 28 days. The concrete mixes differ by the maximum particle size of the clinker powder replacing the binder and the aggregates. The curing conditions are air-dry curing for 28 days and additional water curing for 90 days. The loading conditions consider pre-loading and loading at 28 days, pre-loading at 28 days and loading at 28 + 90 days.

## 2. Experimental Methods

### 2.1. Material Test of Mortar

**Figure 1:**
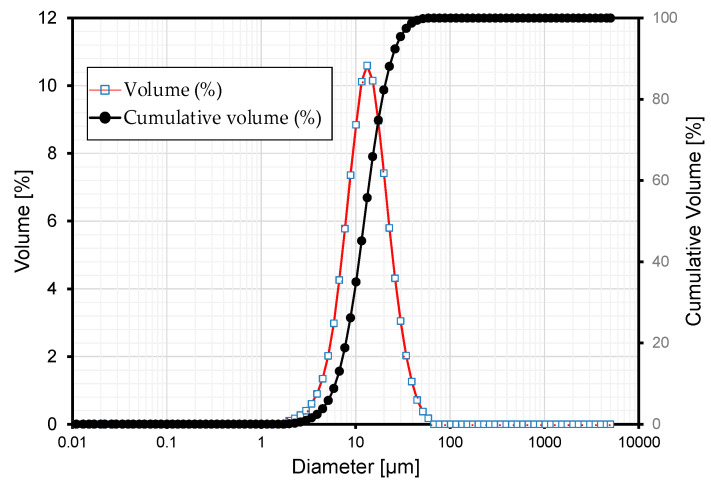
Grain size analysis results of GGBFS.

The first stage of the investigation started with material test on the mortar mixes to be used later in the fabrication of the concrete specimens. Table 1 presents the chemical composition of the raw materials. The grain size analysis of ground granulated blast-furnace slag (GGBFS) revealed grain sizes between 0.011 and 58.953 μm with an average of 10 μm (Figure 1). The grain distribution and chemical composition of GGBFS and clinker appeared to present no significant difference. Table 2 arranges the mix proportions of the 3 series of mortar considered in this study. Plain series is the control series with mortar made of ordinary Portland cement (OPC) and sand. Series 2.5 and 0.85 correspond to mortars with clinker binder and aggregates in which clinker was crushed to have particle sizes of 2.5 mm and 0.85 mm for replacing sand and cement, respectively. A water-to-binder ratio of 0.4 was used in all the mixes.

**Figure 2:**
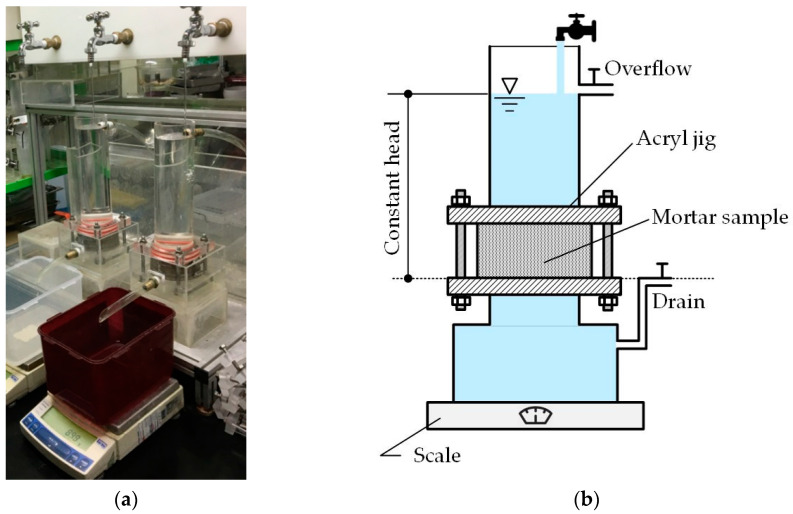
Water flow test: (**a**) photograph of actual test on mortar; (**b**) schematic illustration.

The self-healing performance of pre-cracked mortar specimens was assessed by constant head water flow test (Figure 2) on sets of three ϕ100 × 50 mm cylinders fabricated for each considered mix proportions. The fabricated specimens were stored for 1 day in a constant temperature and humidity chamber at temperature of 20 ± 1 °C and relative humidity of 100% prior to water curing for 27 days at 20 ± 1 °C. Regular cracking was induced by splitting test on the specimens notched in both ends. The crack width was adjusted to 0.25 mm and 0.30 mm by disposing silicon sheets on both ends of the specimens. The cylinders were installed in an acryl mold to fix them during the water flow test. The experimental setup follows the method proposed by Choi et al. [33], which is based on the work of Lepech and Li [34].

### 2.2. Material Test of Concrete

Table 3 summarizes the mix proportions of concrete using the 3 different types of mortar of this study. As mentioned above, two different sizes of clinker powder were considered to replace cement and sand. Among the ingredients, 0.85 stands for clinker binder (particle size < 0.85 mm) and 2.5 for clinker aggregate (grain size < 2.5 mm). SP represents superplasticizer of which the proportion corresponds to 1.5 weight percent.

### 2.3. Test for Fexural Behavior

#### 2.3.1. Test Variables

The three different concrete mixes listed in Table 3 and four different loading and curing conditions were chosen as test variables. The concrete mixes differ by the maximum particle size of the clinker powder replacing the binder and the aggregates: the mix with particle size of 0.85 mm for the clinker replacing aggregates; the mix with 50% of 0.85-mm clinker and 50% of 2.5-mm clinker replacing binder and aggregates; and, the plain series (control). The four loading and curing conditions are:

- V_28D series for the members at 28 days loaded until the ultimate state;
- PLRL_28D series for the members pre-loaded up to 50% of the ultimate load at 28 days followed by loading until the ultimate state;
- RL_28 + 90D series for the members pre-loaded up to 50% of the ultimate load at 28 days, followed by 90 days of water curing prior to loading until the ultimate state;
- V_28 + 90D series for the members loaded up to the ultimate load after 28 days and after 28 + 90 days.

The combination of these test variables gives a total of 12 test members. The members were fabricated with the mixes presented in Table 3 and were subjected to air-dry curing until 28 days. Table 4 arranges the designation and features of the test members. Note that this additional period of 90 days was decided following the work of Alyousif [35], which stated that engineered cementitious composite beams exhibit strength recovery after 90 days of extended moist curing regardless of their size.

#### 2.3.2. Fabrication of Test Members and Test Setup

**Figure 3:**
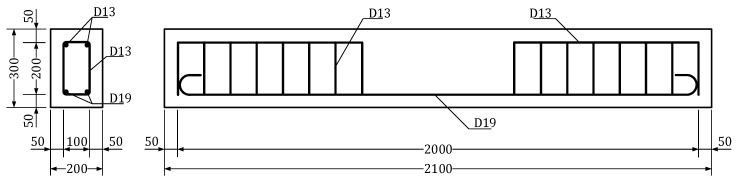
Dimensions and shape of flexural test members.

Figure 3 depicts the shape of the flexural test members for examining the stiffness recovery. The reinforced concrete members present rectangular cross-section with width of 200 mm and height of 300 mm and were designed to have pure bending section of 600 mm. The steel reinforcement uses SD400 bars with a 19 mm-diameter as tensile reinforcement, a 13 mm-diameter as compressive reinforcement and a 13 mm-diameter as shear reinforcement. The materials were first dry-mixed prior to the introduction of water and the admixtures. Wet curing was conducted for two days in a laboratory by covering the members with a curing tent and supplying continuously appropriate humidity. Thereafter, dry-air curing was performed until 28 days.

**Figure 4:**
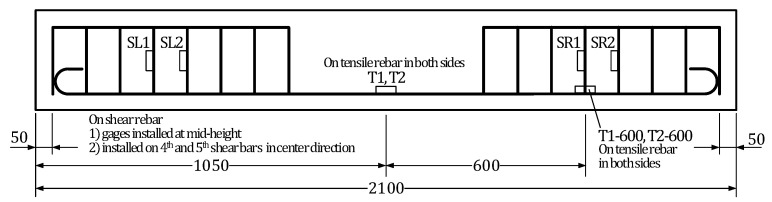
Layout of reinforcement strain gages in test members.

Strain gages were installed on the tensile and shear reinforcing bars before placing the concrete. Four strain gages were disposed on the tensile reinforcement with two sensors at mid-span and two sensors at 1/3 positions, that is at 600 mm in both sides from mid-span. Four strain gages were attached to the shear reinforcement with one sensor in each fourth and fifth shear rebar on the left and right-hand sides of the critical section at mid-span. The layout of the reinforcement strain gages is shown in Figure 4.

**Figure 5:**
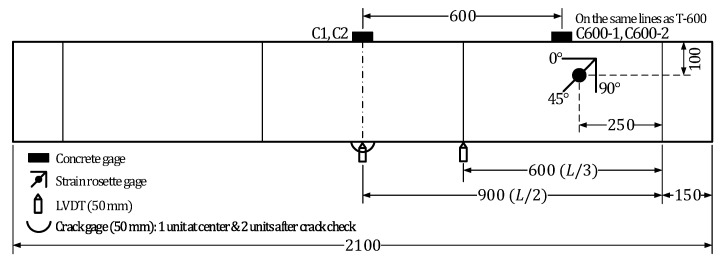
Setup for 4-point loading test.

**Figure 6:**
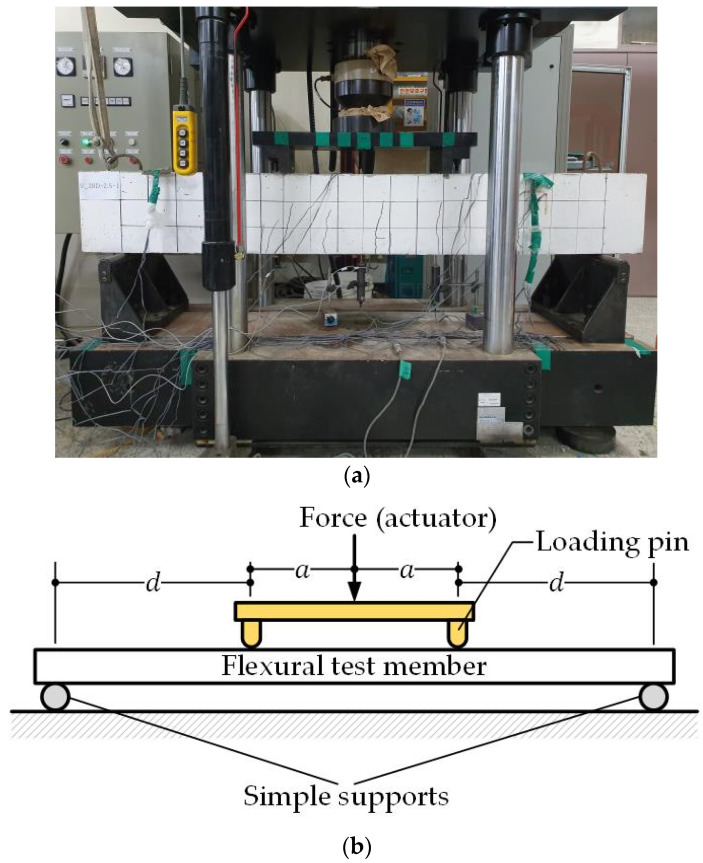
Four-point bending test of flexural members: (**a**) photograph of actual bending test; (**b**) schematic illustration.

Figure 5 shows the setup for the 4-point bending test of the members as prescribed by ASTM C1609. The test members were simply supported by disposing them on supports located 150 mm from the ends of the members to achieve a supported length of 1800 mm. An LVDT was installed at the loading point for measuring the deflection to achieve displacement control for loading applied at speed of 0.5 mm/min. Figure 6 presents a photograph and a schematic illustration of the 4-point bending test of the flexural test members.

**Figure 7:**
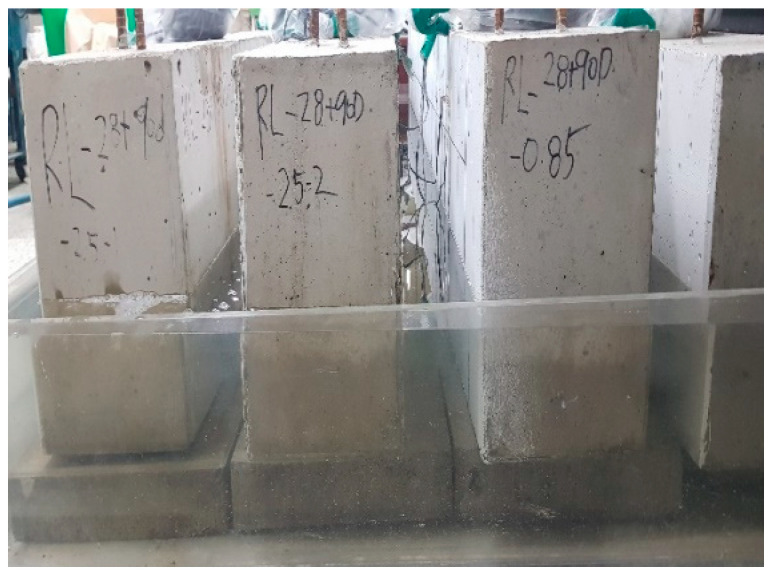
Partial water curing of pre-damaged test members.

As explained in Table 4, pre-loading was applied up to 50% of the ultimate load followed by loading until the ultimate state at 28 days for members PLRL_28D. Members RL_28 + 90D were pre-loaded up to 50% of the ultimate load at 28 days and 150 mm of their lower part was then subjected to partial water curing for 90 days to examine the crack healing performance of pre-damaged members (Figure 7) before loading until the ultimate state. Members V_28 + 90D experienced dry-air curing until 118 days (28 + 90 days) before being loaded up to the ultimate load to examine the stiffness recovery according to the maximum size of the clinker powder replacing the binder and the aggregates.

## 3. Test Results

### 3.1. Material Test Results

The material test results of mortar and concrete are presented. Table 5 shows the compressive strength and slump flow measured for each of the considered mortars listed in Table 2. In Table 5, the values in parentheses represent the standard deviation, σ.

**Figure 8:**
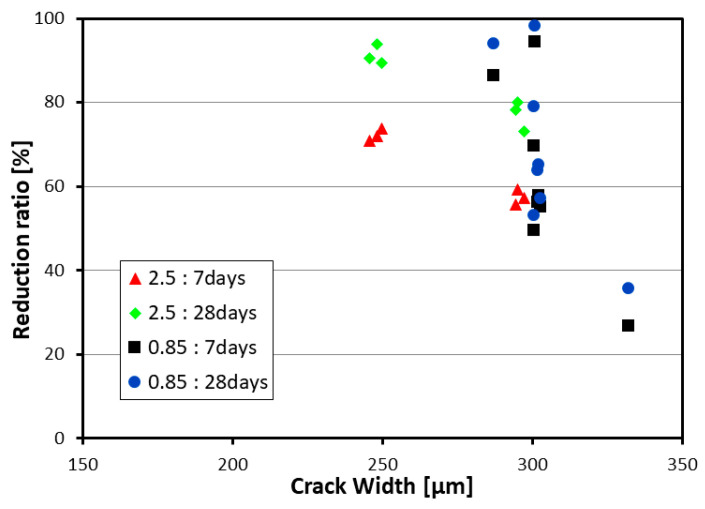
Water flow test results.

The water flow test results are plotted in Figure 8. The reduction ratio in Figure 8 indicates the water flow at 7 or 28 days as compared to that at day zero. This reduction ratio appears to be smaller as the crack width is large and the age is young. On the whole, the reduction ratio of the 0.85-mm series is higher than that of the 2.5-mm series samples, which shows that the replacement of binder by clinker powder brings a greater crack healing effect.

The basic physical properties of the concrete mixes are presented in Table 6 and Table 7. Recalling that some test members experienced pre-damage at 28 days and were reloaded 90 days later, measurement of the physical properties was conducted at 28 days and 28 + 90 days. In Table 5, the compressive strength, *f_ck_*, was measured on prismatic specimens at 28 days and 90 days later in compliance with KS L ISO 679. Similar levels of compressive strength were developed in all the mixes. The elastic modulus, *E_c_*, was measured as the slope of the linear 10–40% region in the stress–strain diagram using triaxial concrete strain rosette gages attached to the ϕ100 × 200-mm mold. Results similar to those of the compressive strength were obtained. The 0.85-mix series with a relatively meaningful binder replacement ratio by 0.85 mm of clinker exhibited the best physical properties. The slump and air content measured on fresh concrete are also indicated in Table 6. Table 7 arranges the flexural strength, *f_b_*, measured using the third-point loading method in compliance with KS F 2048. In Table 7, the values in parentheses represent the standard deviation, σ. Similarly to the results of Table 6, similar flexural strength was measured in all the mixes. Here also, the 0.85-mix series with a relatively meaningful binder replacement ratio by 0.85 mm of clinker exhibited the best physical properties. In Table 6 and Table 7, the values in parentheses represent the standard deviation, σ.

### 3.2. Flexural Test Results

#### 3.2.1. Crack, Yield, Ultimate Loads and Failure Pattern

All the test members exhibited negligible difference in the crack, yield and ultimate loads apart from some slight variability. Moreover, all the test members failed through flexure. Table 8 summarizes the measured crack, yield and ultimate loads as well as the failure pattern of the 12 test members listed in Table 4.

#### 3.2.2. Load-Deflection Relations

**Figure 9:**
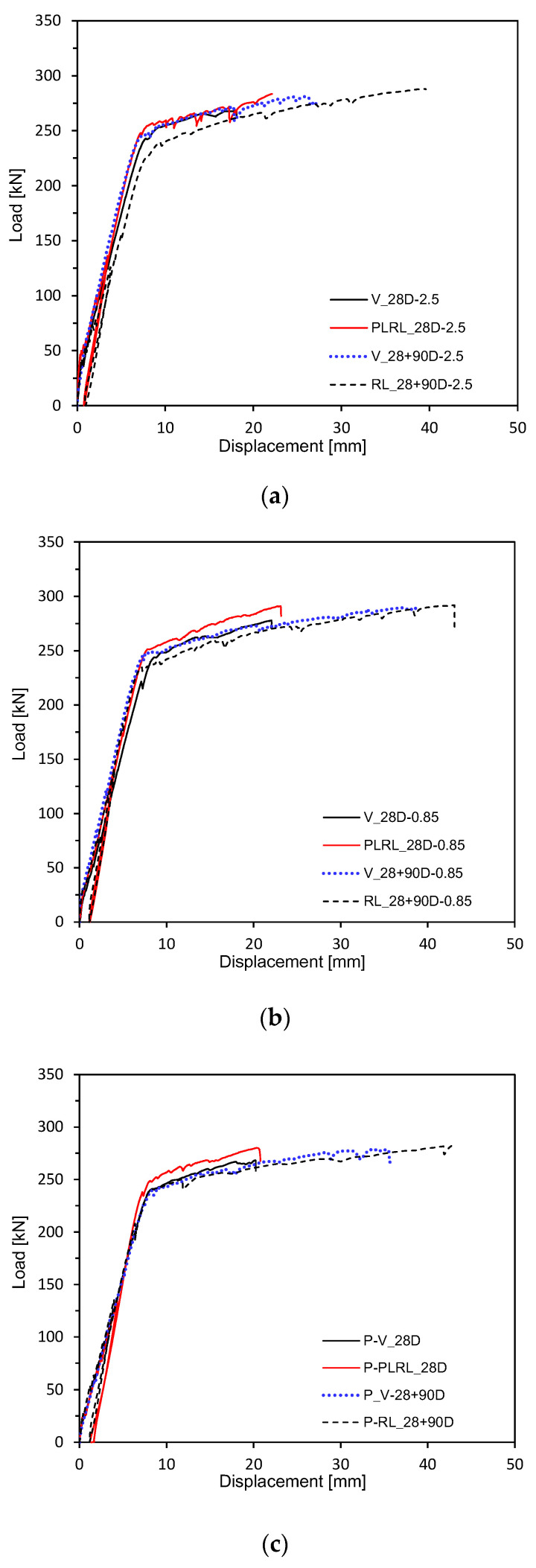
Load-deflection measurement by mixes: (**a**) 2.5 series; (**b**) 0.85 series; (**c**) Plain series.

**Figure 10:**
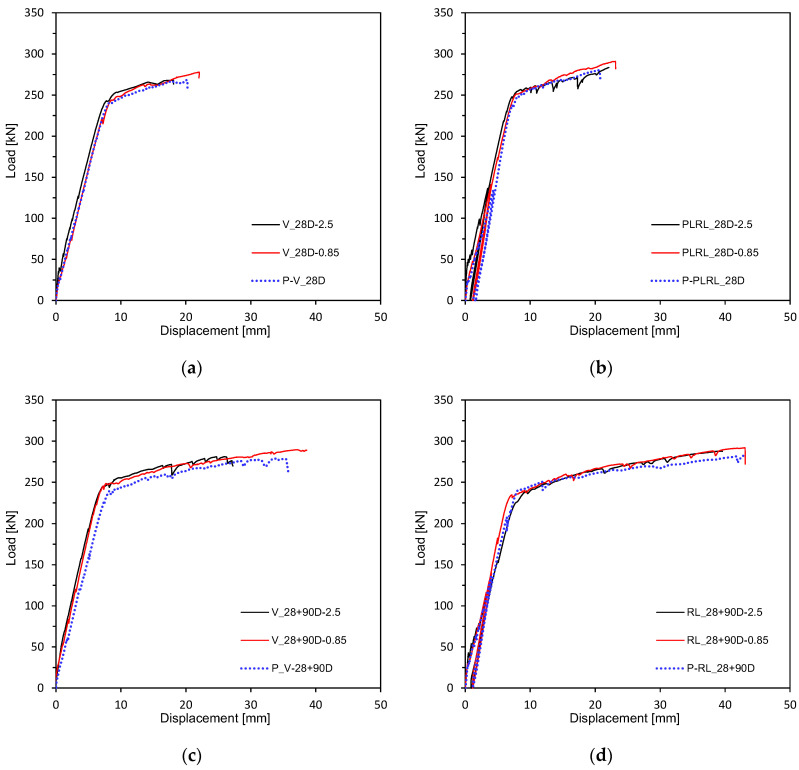
Load-deflection measurement by loading conditions: (**a**) V_28D series; (**b**) PLRL_28D series; (**c**) RL_28D series; (**d**) RL_28 + 90D series.

**Figure 11:**
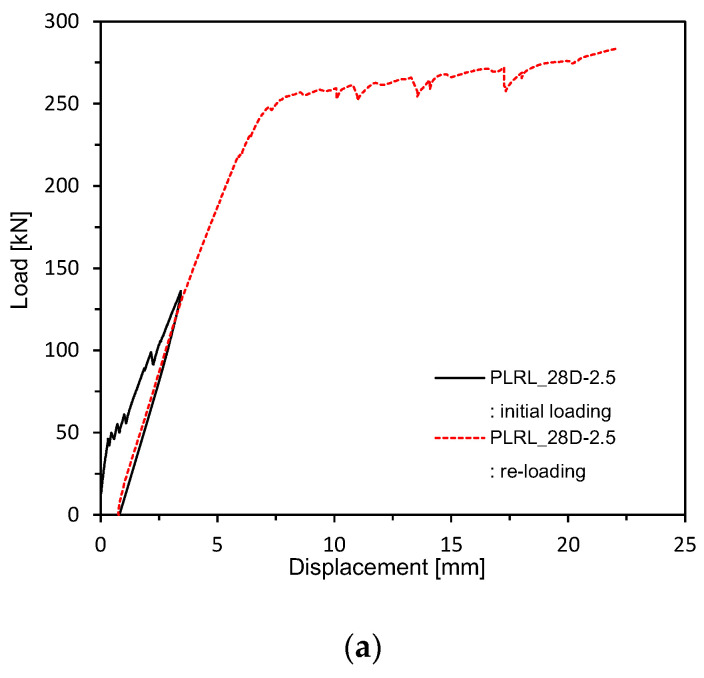

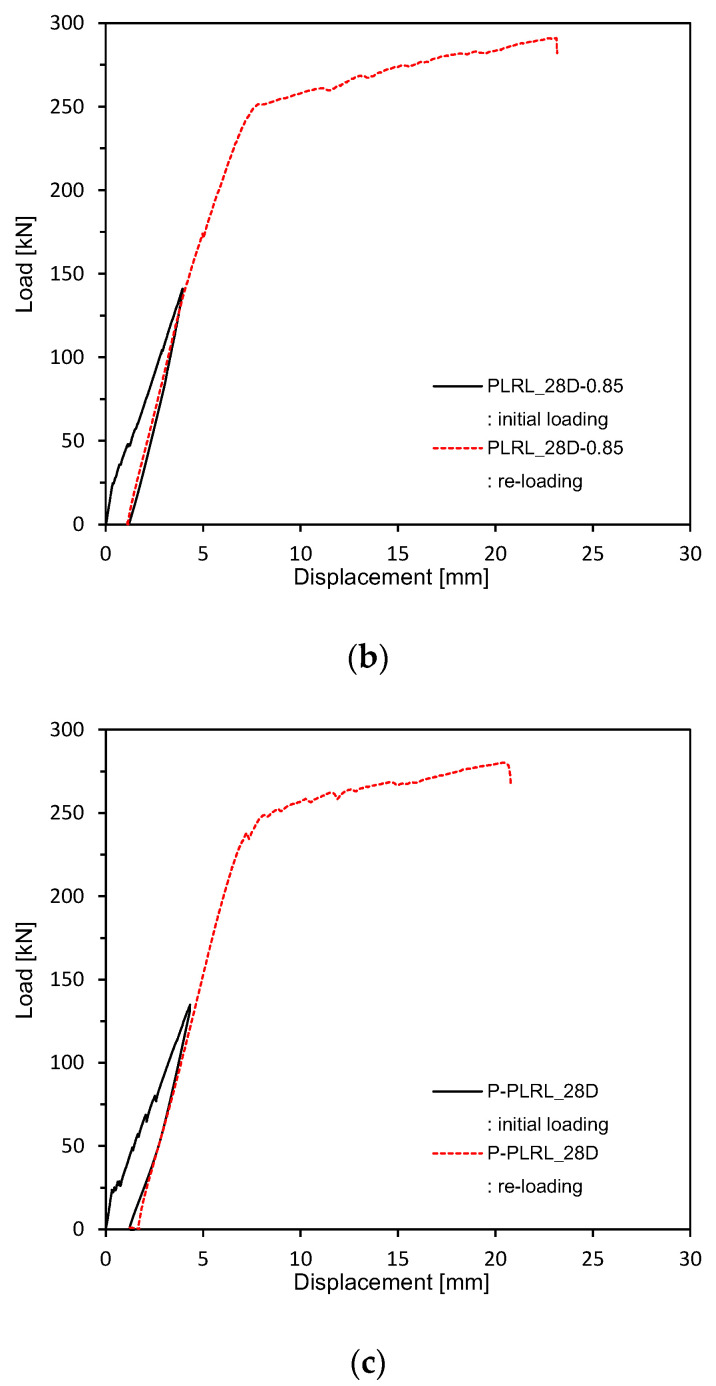
Load-deflection measurement after 28 days (up to ultimate load after pre-loading): (**a**) 2.5 series; (**b**) 0.85 series; (**c**) Plain series.

**Figure 12:**
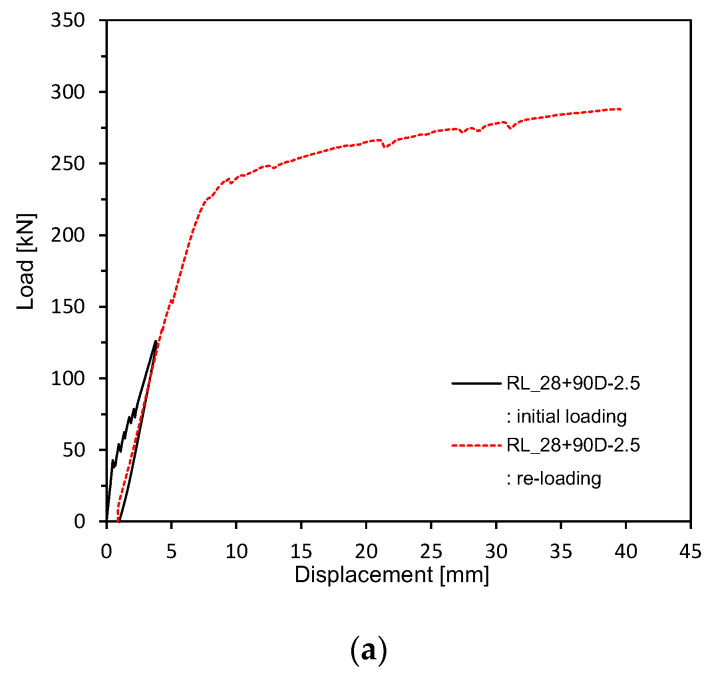

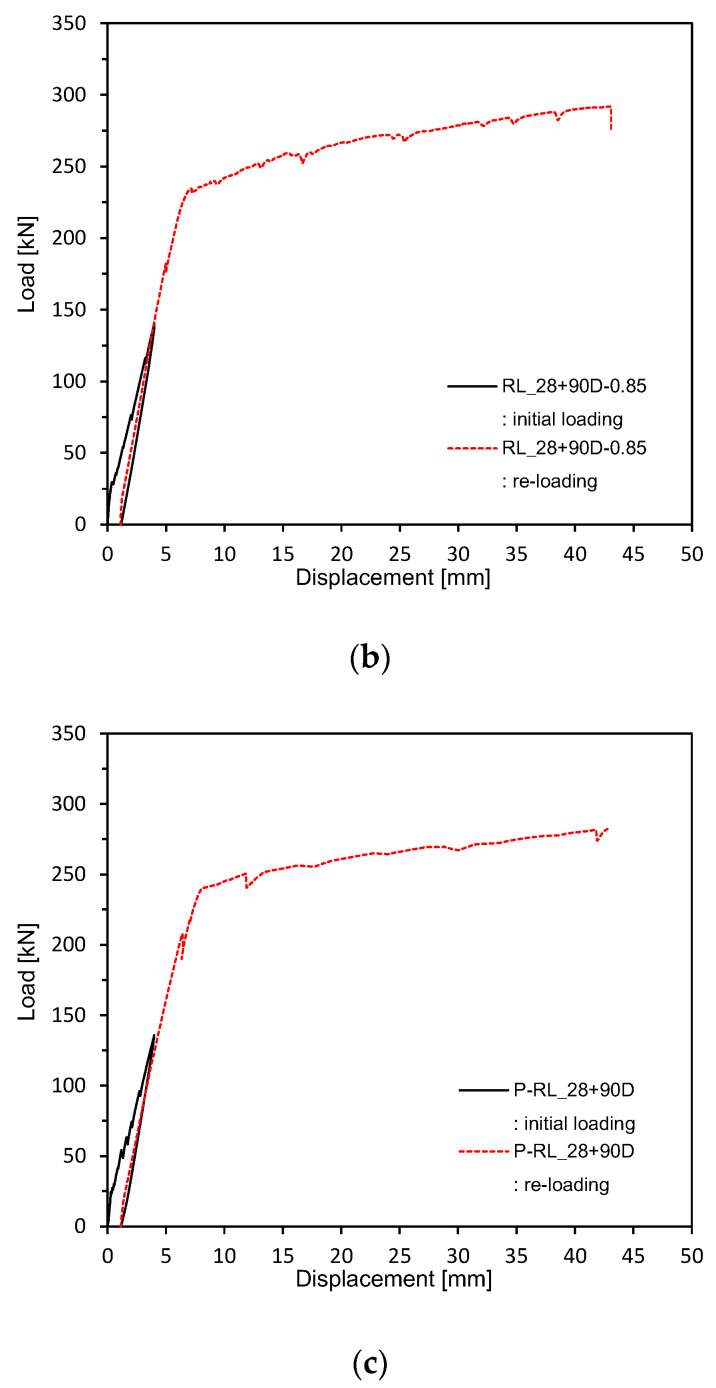
Load-deflection measurement after 28 + 90 days (up to ultimate load after pre-loading and additional 90 days of curing after pre-loading): (**a**) 2.5 series; (**b**) 0.85 series; (**c**) Plain series.

Figure 9 and Figure 10 plot the load-deflection relations measured after 28 days of curing. Figure 11 shows the load-deflection relations after pre-loading. Figure 12 plots the load-deflection relations after pre-loading and additional 90 days of curing. All the test members show linear load-deflection relations until early cracking followed by a nonlinear increase in the deflection after cracking and finally the increase in the load until the ultimate load. The members that experienced additional 90 days of curing after 28 days appear to undergo ultimate deflection nearly larger than 100% of that of the members loaded after 28 days of curing due to the plastic deformation effect induced by their age and the yield of steel reinforcement. This result confirms the increasing trend in the flexural resistance observed by Alyousif [35].

#### 3.2.3. Load–Rebar Strain Relations

**Figure 13:**
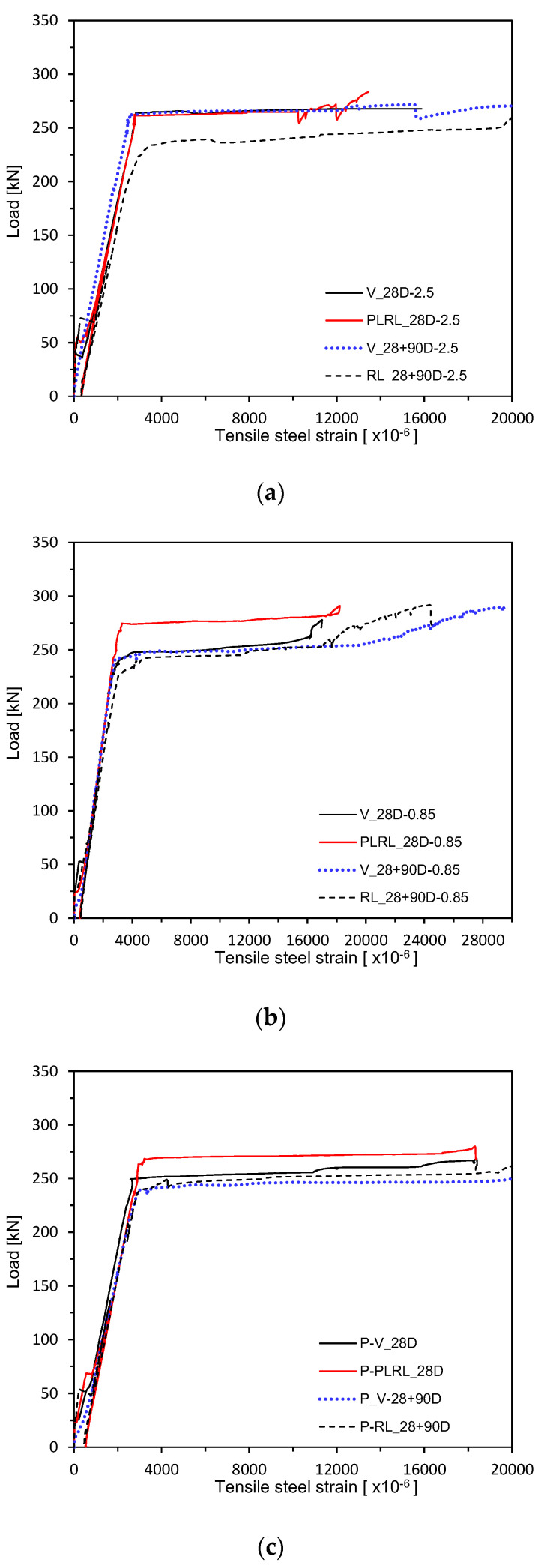
Load–steel reinforcement strain measurement by mixes: (**a**) 2.5 series; (**b**) 0.85 series; (**c**) Plain series.

**Figure 14:**
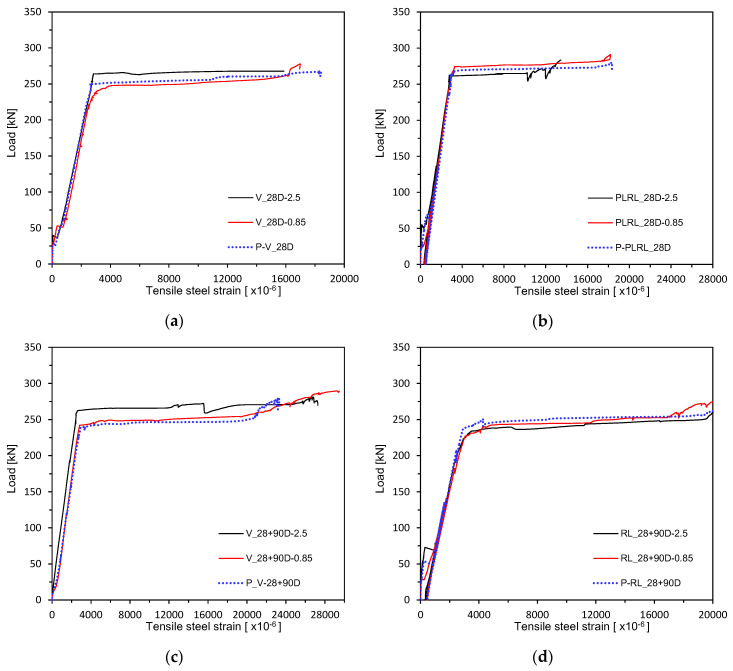
Load–steel reinforcement strain measurement by loading conditions: (**a**) V_28D series; (**b**) PLRL_28D series; (**c**) RL_28D series; (**d**) RL_28 + 90D series.

**Figure 15:**
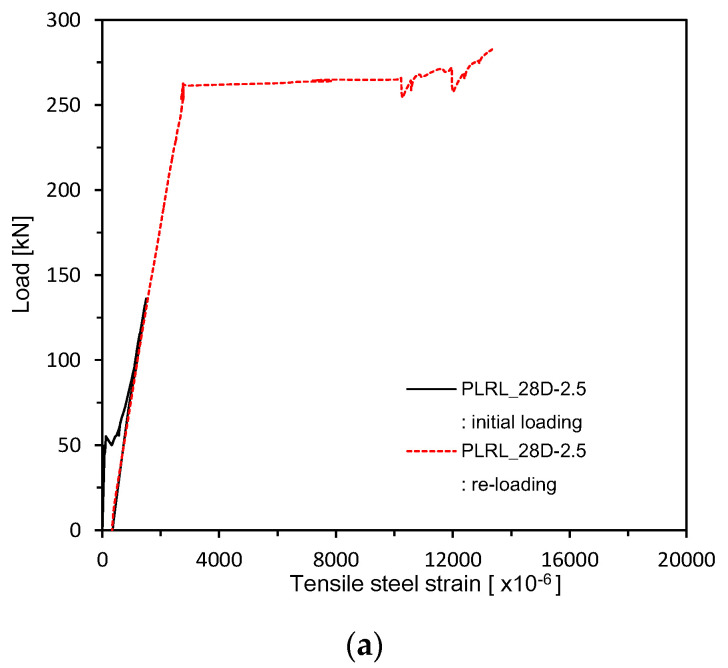

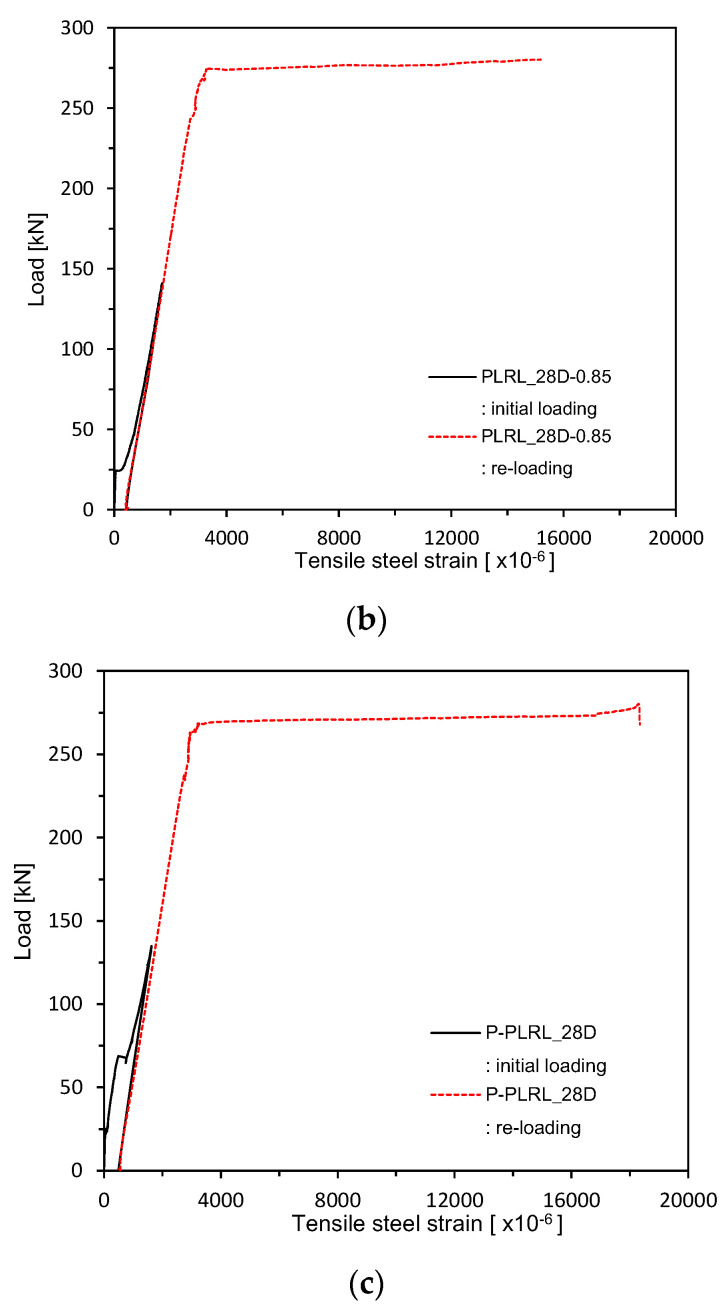
Load–steel reinforcement strain measurement after 28 days (up to ultimate load after pre-loading): (**a**) 2.5 series; (**b**) 0.85 series; (**c**) Plain series.

**Figure 16:**
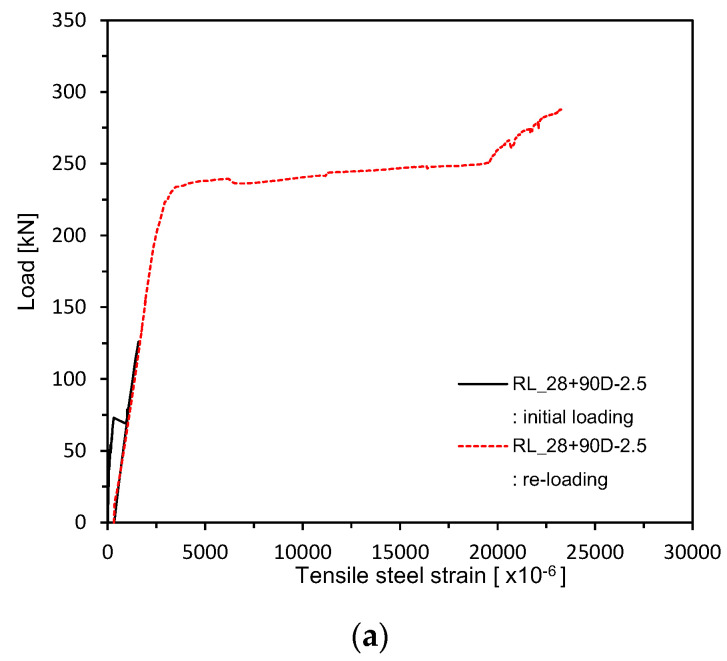

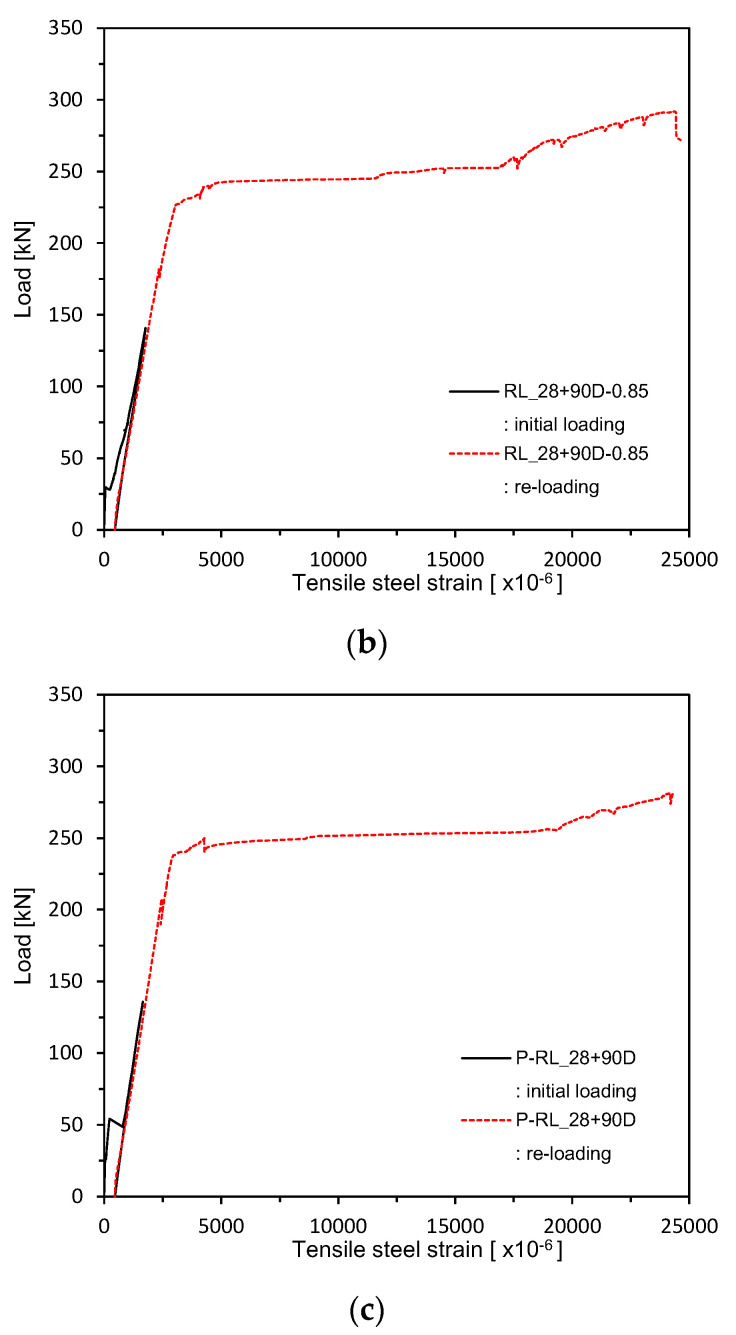
Load–steel reinforcement strain measurement after 28 + 90 days (up to ultimate load after pre-loading and additional 90 days of curing after pre-loading): (**a**) 2.5 series; (**b**) 0.85 series; (**c**) Plain series.

Figure 13 and Figure 14 plot the load–rebar strain relations measured after 28 days of curing. Figure 15 shows the load–rebar strain relations after pre-loading. Figure 16 plots the load–rebar strain relations after pre-loading and an additional 90 days of curing. Here, the rebar strain practically did not develop before cracking and started to increase linearly after the initiation of cracks to increase starkly after yielding.

#### 3.2.4. Load–Concrete Strain Relations

**Figure 17:**
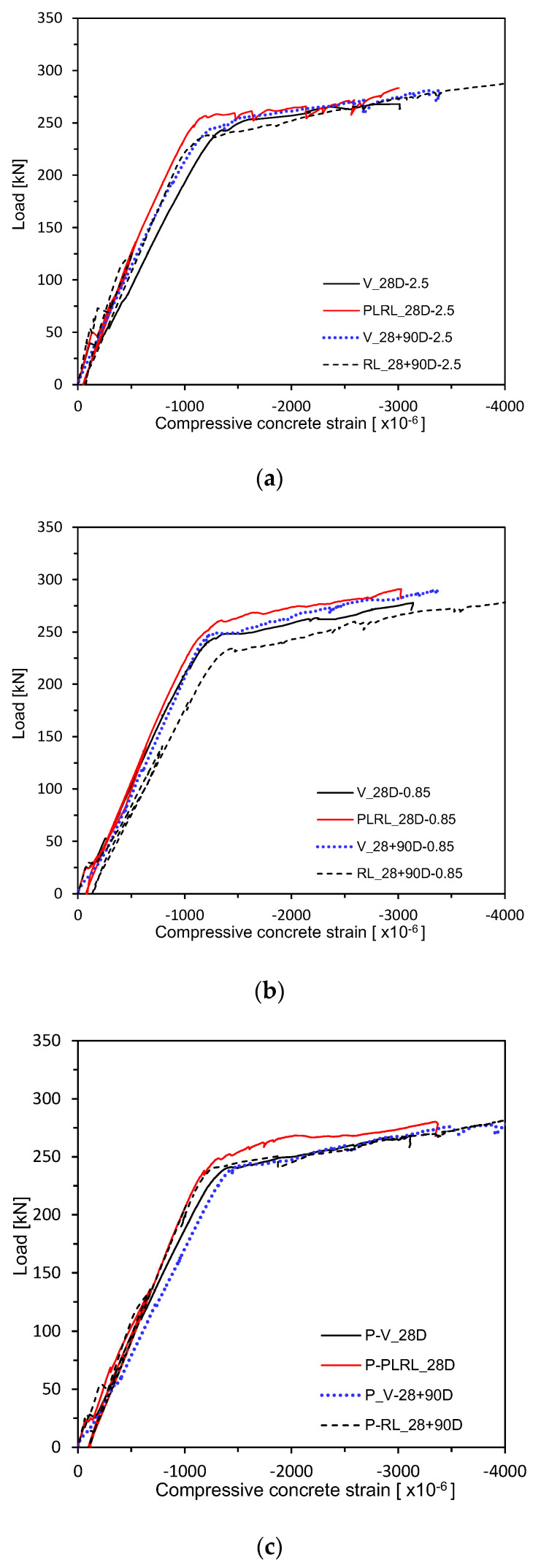
Load–concrete strain measurement by mixes: (**a**) 2.5 series; (**b**) 0.85 series; (**c**) Plain series.

**Figure 18:**
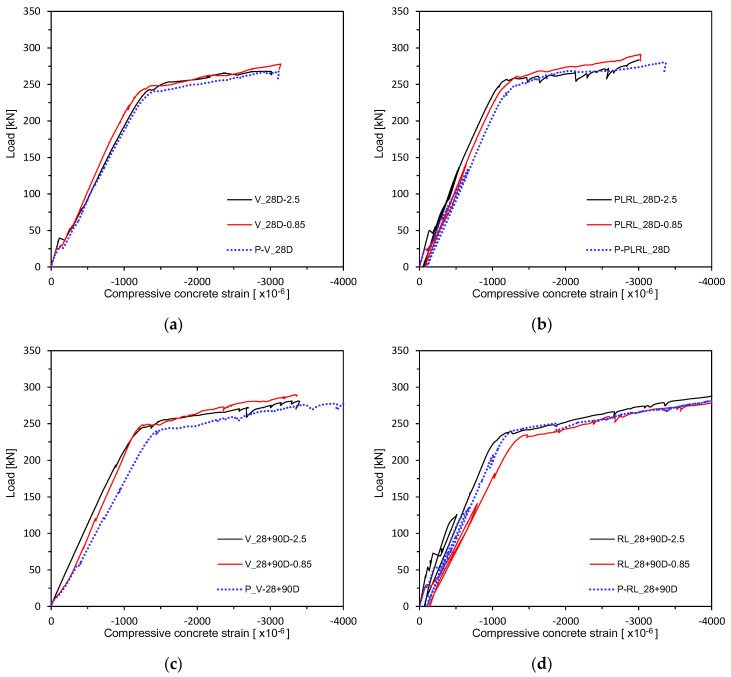
Load–concrete strain measurement by loading conditions: (**a**) V_28D series; (**b**) PLRL_28D series; (**c**) RL_28D series; (**d**) RL_28 + 90D series.

**Figure 19:**
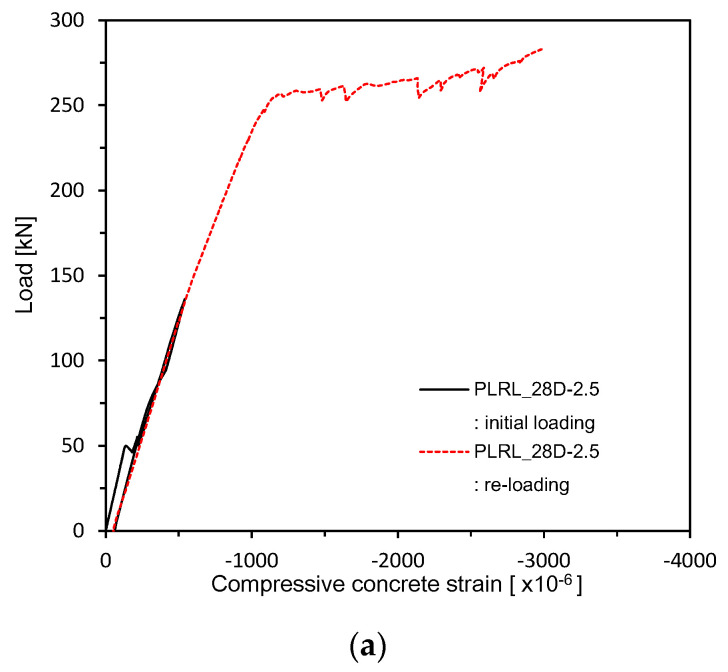

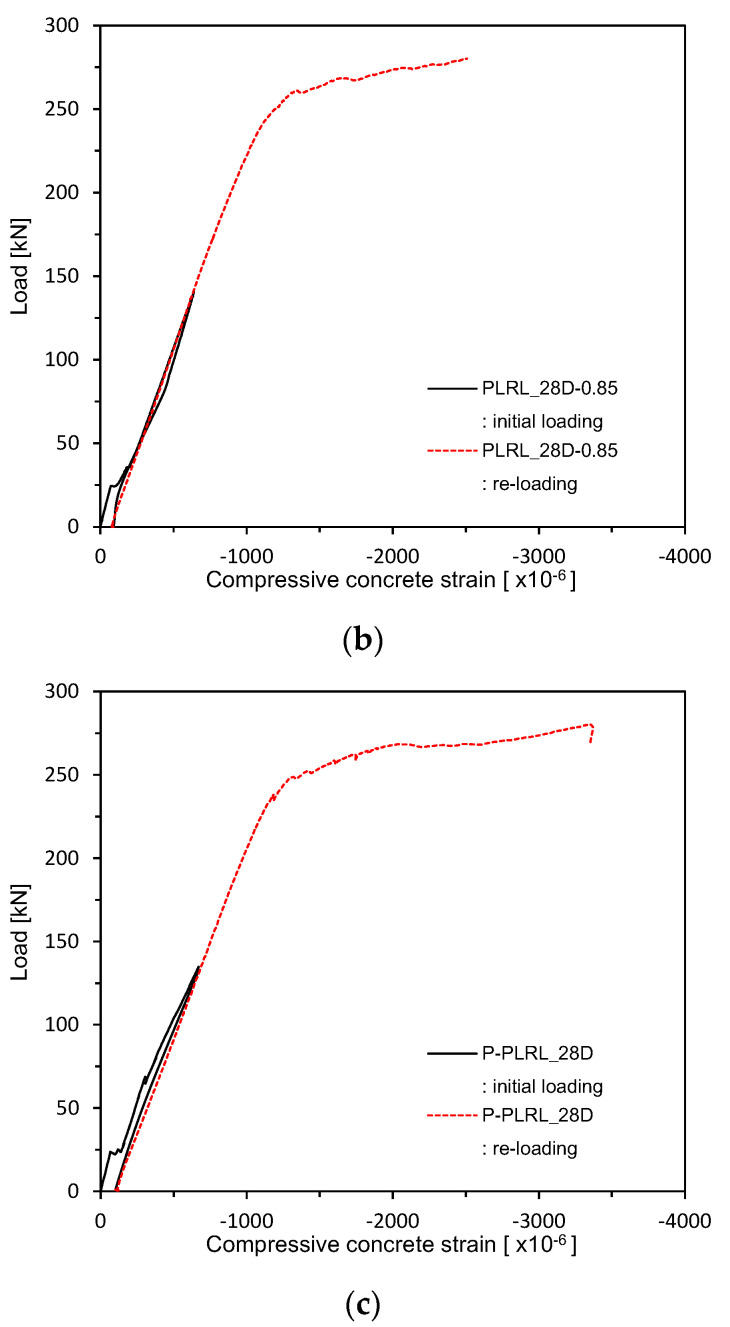
Load–concrete strain measurement after 28 days (up to ultimate load after pre-loading): (**a**) 2.5 series; (**b**) 0.85 series; (**c**) Plain series.

**Figure 20:**
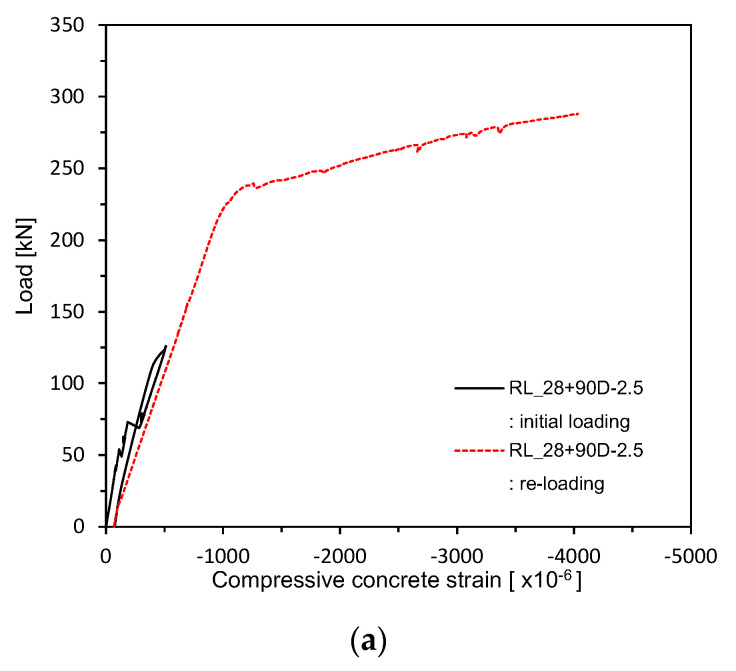

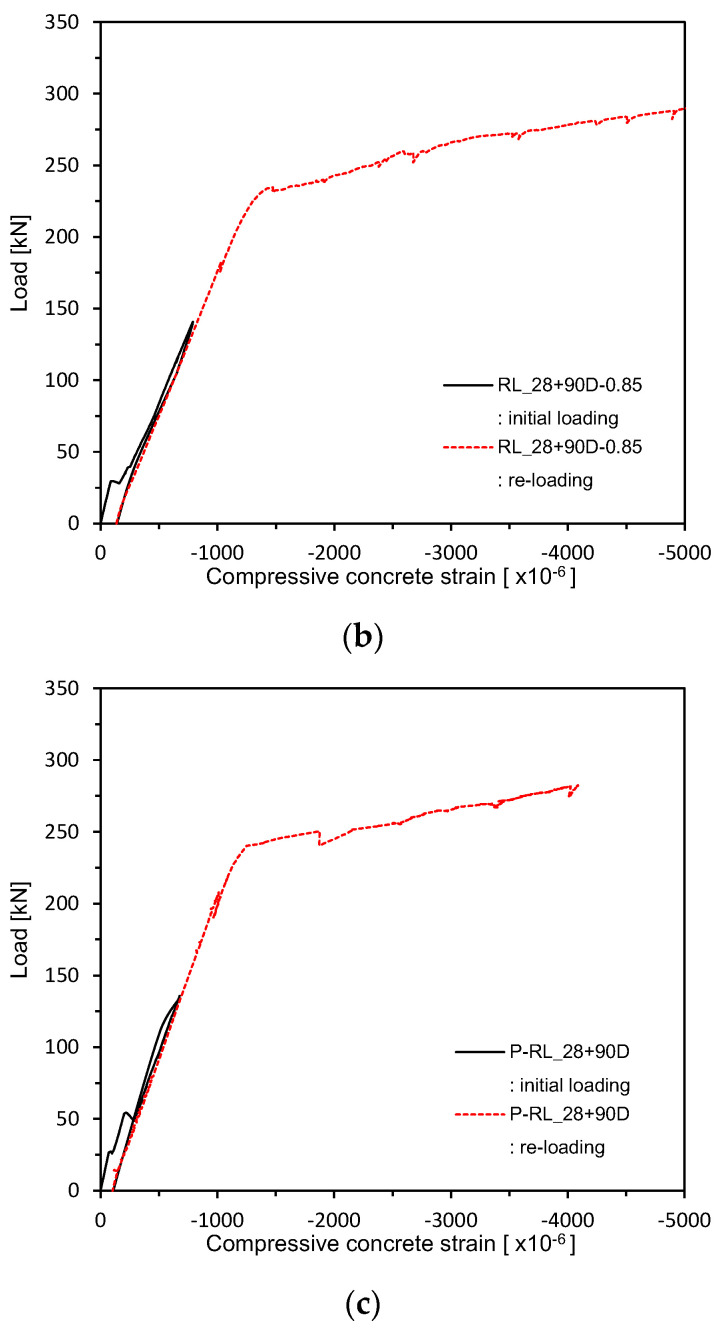
Load–concrete strain measurement after 28 + 90 days (up to ultimate load after pre-loading and additional 90 days of curing after pre-loading): (**a**) 2.5 series; (**b**) 0.85 series; (**c**) Plain series.

Figure 17 and Figure 18 plot the load–concrete strain relations measured after 28 days of curing. Figure 19 shows the load–concrete strain relations after pre-loading. Figure 20 plots the load–concrete strain relations after pre-loading and additional 90 days of curing. The members loaded up to the ultimate at 28 days developed ultimate concrete strain of about 0.0030 but those which experienced additional 90 days of curing saw their ultimate concrete strain be around 0.0040 due to effects of aging and residual deformation.

#### 3.2.5. Crack Patterns

**Figure 21:**
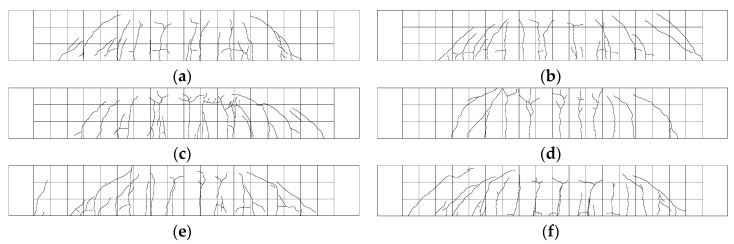

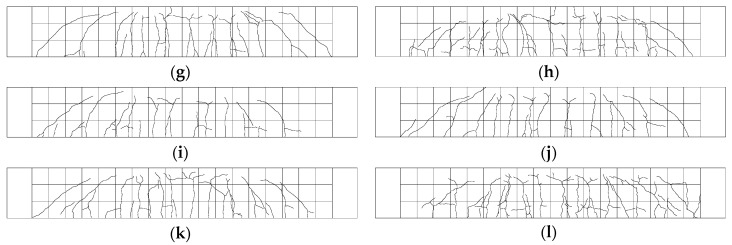
Crack patterns of test members: (**a**) V_28D-2.5; (**b**) PLRL_28D-2.5; (**c**) RL_28 + 90D-2.5; (**d**) V_28 + 90D-2.5; (**e**) V_28D-0.85; (**f**) PLRL_28D-0.85; (**g**) RL_28 + 90D-0.85; (**h**) V_28 + 90D-0.85; (**i**) P-V_28D; (**j**) P-PLRL_28D; (**k**) P-RL_28+90D; (**l**) P-V_28 + 90D.

The number of vertical cracks observed in the test members ran between 10 and 15 with 11 cracks for member V_28D-2.5, 15 for member PLRL_28D-2.5, 14 for member RL_28 + 90D-2.5, 10 for member V_28 + 90D-2.5, 13 for member V_28D-0.85, 14 for member PLRL_28D-0.85, 12 for member RL_28 + 90D-0.85, 15 for member V_28 + 90D-0.85, 13 for member P-PLRL_28D, 14 for member P-RL_28 + 90D, and 13 for member P-V_28 + 90D. The crack pattern after the flexural test of the test members is shown in Figure 21. All the members experienced flexural cracking and flexural failure.

## 4. Discussion of Results

### 4.1. Visual Observation of Crack Healing Effect

**Figure 22:**
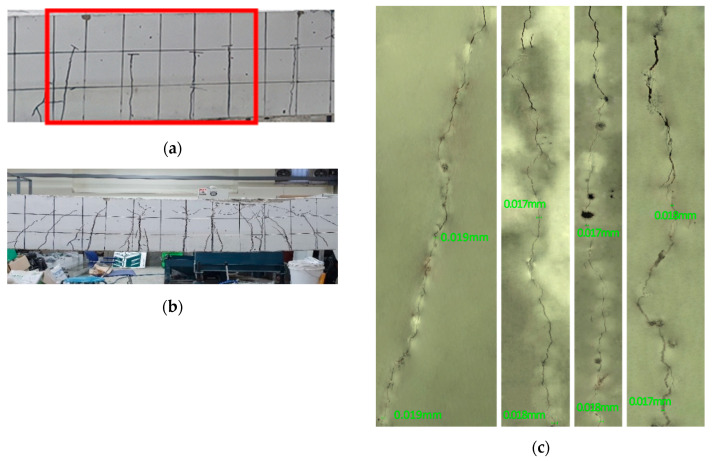
Crack healing of member RL_28 + 90D-2.5: (**a**) after pre-loading at 28 days + 90 days of additional curing; (**b**) after loading at 28 + 90 days; (**c**) enlargement of cracks after completion of test.

**Figure 23:**
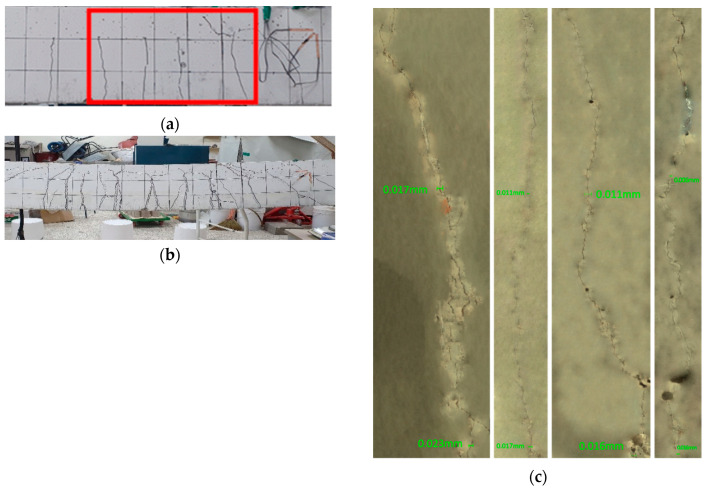
Crack healing of member RL_28 + 90D-0.85: (**a**) after pre-loading at 28 days + 90 days of additional curing; (**b**) after loading at 28 + 90 days; (**c**) enlargement of cracks after completion of test.

**Figure 24:**
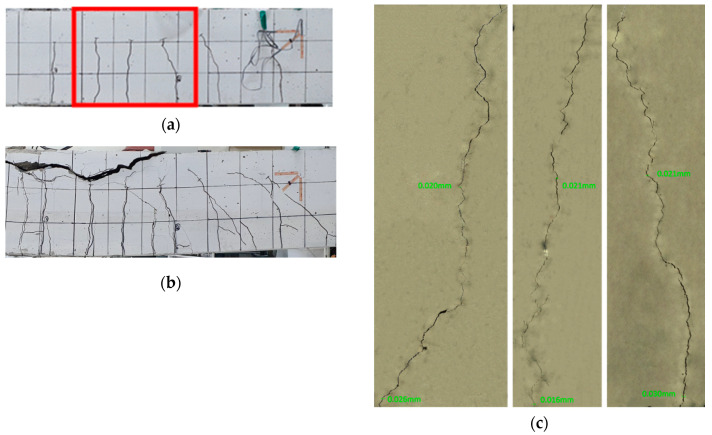
Crack healing of member P-RL_28 + 90D: (**a**) after pre-loading at 28 days + 90 days of additional curing; (**b**) after loading at 28 + 90 days; (**c**) enlargement of cracks after completion of test.

Figure 22, Figure 23 and Figure 24 illustrate the cracks and healing of the test members after pre-damage at 28 days and after the additional 90 days of curing. The target crack width before loading of the members pre-loaded up to 50% of the ultimate load was 0.16 mm. In view of the figures, the maximum crack width after 90 days of additional curing ranged between 0.019 and 0.053 mm. Such a result can be attributed to various factors like the crack closure generated by the removal of the load and the autogenous healing.

The enlarged images of the cracks reveal that the 0.85-series members replacing binder by 100% of clinker achieved the best crack healing performance. The 2.5-series members replacing each binder and aggregates by 50% of clinker exhibited crack healing performance in-between that of the P-series members without clinker and that of the 0.85-series members.

An attempt to objectively quantify the crack healing effect is achieved by measuring the crack distribution density from the observed crack patterns shown in Figure 21. Table 9 arranges the crack distribution density of the flexural specimens obtained by counting the number of cracks at the bottom of the members and dividing this number by the member’s length (2000 mm). All the specimens experienced a similar number of vertical cracks. Moreover, the overall crack pattern observed in the beam members were similar to those of the experimental results reported by Alyousif [35].

A closer look reveals that, for the 0.85-series members, the RL-series members that suffered pre-damage exhibit relatively smaller crack density than the V-series members which did not experience pre-damage. Besides, the opposite occurs for the P-series and 2.5-series members. This situation can be credited to the fact that, compared to the 2.5-series members, the 0.85-series members exhibit a strong healing effect for cracks thinner than 200 μm, which reduces the number of cracks, but does not heal wider cracks, which experience crack opening under reloading. This phenomenon becomes more acute in older members. According to the studies by Sunayana and Barai [36] and Sturm et al. [37], the closer cracking spacing in the beams might be caused by the effect of high shrinkage in beams. In other words, the healing effect improves with time. Consequently, the fine grain size of clinker makes it possible to replace fine aggregates and longer healing time increases the crack healing effect.

### 4.2. Evaluation of Crack Healing Effect by Water Flow Test

**Figure 25:**
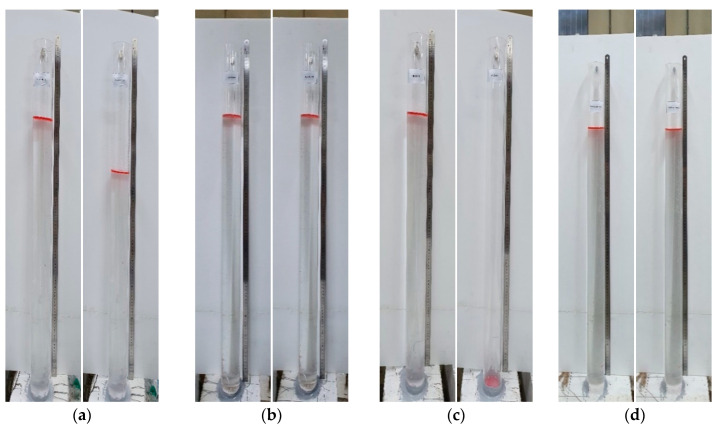
Results of water flow test: (**a**) 2.5 series; (**b**) 0.85 series; (**c**) Plain series; (**d**) healthy part.

The crack healing effect was also examined by water flow test. Figure 25 shows the water flow test performed to evaluate quantitatively the crack healing effect achieved in the test members after pre-damage at 28 days and an 90 additional days of curing. The water flow test was performed using cylindrical tubes made of acryl with a diameter of 100 mm and height of 1000 mm. The tubes were installed over the cracked and healthy parts of the members that were waterproofed. Water was filled in the tubes up to the specified height and the difference in water level in the tube was measured after 4 h.

The results of the water flow test are arranged in Table 10. There is no way to provide direct figures about the crack healing effect but the drop of the water level in the water flow test can give indirect insight on this effect. It appears that the 0.85-series members replacing binder by 100% of clinker achieved the best crack healing performance whereas the 2.5-series members replacing each binder and aggregates by 50% of clinker exhibited crack healing performance in-between that of the P-series members without clinker and that of the 0.85-series members. This verifies the previous observation done in view of the enlarged images of the cracks.

The results arranged in Table 10 support the conclusion of Escoffres et al. [12], although it concerned high-performance fiber reinforced concrete, that the addition of crystalline admixture slowed down the self-healing process measured by water permeability but provided greater mechanical recovery under tension to the material. In other words, this means that the improvement of durability by self-healing will remain in presence of crystalline admixture until a significant variation of the applied load.

### 4.3. Evaluation of Crack Healing Effect on Mechanical Performance

#### 4.3.1. Yield and Ultimate Loads

**Figure 26:**
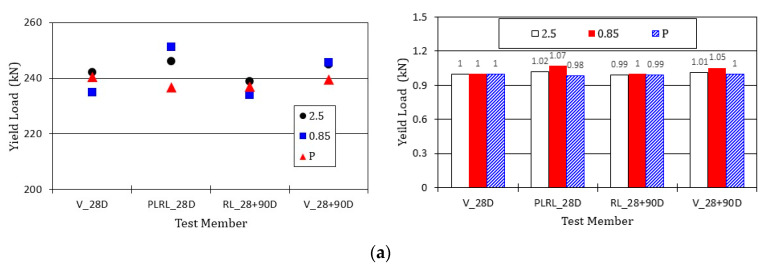

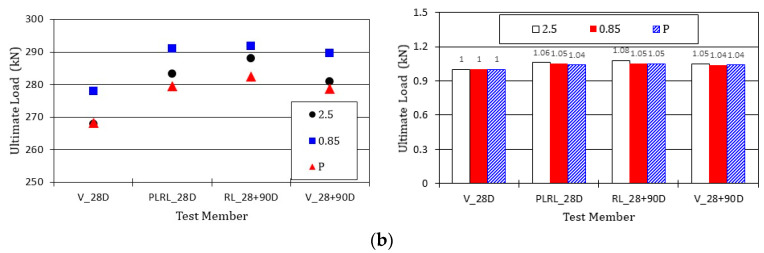
Comparison of reinforcement yield load and members’ ultimate load: (**a**) yield load of steel reinforcement; (**b**) ultimate load of test members.

Figure 26 compares the yield load of the steel reinforcement and the ultimate load of the members after pre-damage at 28 days. All the test members exhibit similar tendency. Compared to member V_28D which experienced 28 days of air-dry curing and loaded up to the ultimate state at 28 days, the test members showed a maximum difference of 7% for the yield load and approximately 8% for the ultimate load. Considering that the difference in the load was 4% to 5% between 28 days and 28 + 90 days as well as the corresponding difference in the strength, the P-series members without crack healing agent seem to undergo an actual difference of 2% to 3% in the yield and ultimate load with the same tendency as those observed in the other members. This last result may indicate that the crack healing effect has practically no effect in improving the yield and ultimate loads of the reinforced concrete member.

#### 4.3.2. Energy Absorption Capacity and Ductility Index

**Figure 27:**
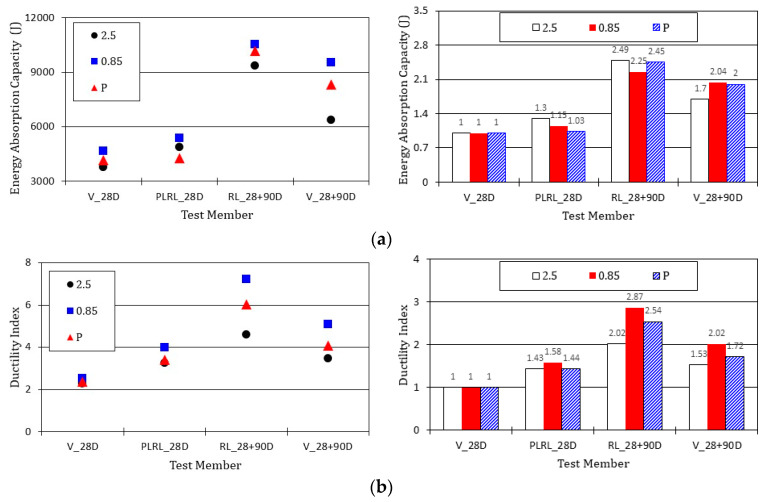
Comparison of energy absorption capacity and ductility index: (**a**) energy absorption capacity; (**b**) ductility index.

Figure 27 compares the energy absorption capacity and ductility index of the test members after pre-damage at 28 days. It appears that the energy absorption capacity increased with the age. Member RL_28 + 90D, which was pre-damaged and experienced additional curing for 90 days, exhibited the highest energy absorption capacity. Such an increase in the energy absorption capacity can be attributed to the increase in the concrete strength with the age and the continuous evolution of cementitious composites with time [34]. Moreover, the members that were pre-damaged and reloaded developed higher energy absorption capacity because of the relatively larger displacement sustained during reloading due to the rebar yield and plastic deformation induced by pre-damage compared to that of the not pre-damaged members. The ductility index of all the test members shows a tendency resembling that of the energy absorption capacity. Compared to member V_28D, the difference in the energy absorption capacity reached a maximum of 249% and that for the ductility index reached approximately 287%. The crack healing had some effect on the increase in the energy absorption capacity and the ductility index but this increasing effect appears to be minimal when considering the increase in the energy absorption capacity brought by the age and the larger displacement sustained during reloading caused by the plastic deformation induced by pre-damage.

## 5. Conclusions

The present study examined the effect of self-healing on the flexural behavior of reinforced concrete members. The considered healing agents were clinker binder and aggregates as well as crystalline admixtures. A total of 12 test members were fabricated with respect to 3 different concrete mixes and 4 different loading and curing conditions. The following conclusions can be drawn from the experimental results.

1. The utilization of mineral admixtures was seen to improve the self-healing performance of mortar. Especially, healing ratios of 100% for crack widths smaller than 200 μm and 85% to 90% for crack widths of 250 μm were observed according to the admixing of clinker binder and aggregates. All the considered mixes exhibited similar results in terms of the compressive strength, elastic modulus and flexural strength. The series with a relatively meaningful binder replacement ratio by 0.85-mm clinker exhibited the best physical properties.
2. A negligible difference was observed in the crack, yield and ultimate loads. Failure occurred through flexure with a similar number of vertical cracks. All the test members showed linear load-deflection relationship until the initiation of cracks followed by a nonlinear increase in the deflection beyond the crack initiation and the load continued to increase until the ultimate load. For the test members cured additionally for 90 days after 28 days, the ultimate deflection reached more than 100% of that of the members loaded after 28 days of curing due to the plastic deformation caused by the yield of the rebar and the aging.
3. Rebar strain practically did not occur prior to the initiation of cracks and grew linearly after cracking to experience a large increase after yielding. The ultimate concrete strain in the members loaded up to the ultimate load at 28 days reached 0.0030 but that in the members that were additionally cured for 90 days after 28 days reached 0.0040 due to the aging and the effect of residual deformation.
4. The maximum crack width observed after 90 days of additional curing ranged between 0.019 and 0.053 mm. These values can be attributed to various factors like crack closure following the removal of loading as well as crack healing. The enlarged images of the cracks revealed that the 0.85-series members replacing binder by 100% of clinker achieved the best crack healing performance. The 2.5-series members replacing each binder and aggregates by 50% of clinker exhibited crack healing performance in-between that of the P-series members without clinker and that of the 0.85-series members. This observation was confirmed by the results of the water flow test performed on the test members.
5. In terms of mechanical performance, the crack healing property of concrete increased, to some extent, the rebar yield load, the members’ ultimate load and energy absorption capacity and ductility index. However, this effect appeared to be minimal when considering the increase in the energy absorption capacity brought by the age and the larger displacement sustained during reloading caused by the plastic deformation induced by pre-damage.
6. An attempt to objectively quantify the crack healing effect by measuring the crack distribution density from the observed crack patterns was performed and this confirmed the crack healing effect provided by clinker powder.
7. Finally, the fine grain size of clinker made it possible to replace fine aggregates and the longer healing time increased the crack healing effect.

## Figures and Tables

**Table 1:** Chemical composition of raw materials (in weight ratio).

Material	CaO	SiO_2_	Al_2_O_3_	Fe_2_O_3_	MgO	K_2_O	Na_2_O	SO_3_
GGBFS	42.51	29.13	15.82	0.67	4.43	0.52	0.28	3.59
Clinker	64.34	22.87	4.96	2.94	1.28	0.82	0.23	0.44

**Table 2:** Mix proportions of mortar (in kg/m^3^, W/B = 0.4).

Series	Binder					Aggregate	
	OPC	GGBFS	Na_2_SO_4_	Anhydrite	Clinker Binder	Clinker Sand	Sand
Plain	690	-	-	-	-	-	1380
2.5	428	173	10	10	69	69	1311
0.85	428	173	10	10	138	-	1311

**Table 3:** Mix proportions of concrete (in kg/m^3^).

Series	Water	OPC	GGBFS	Na_2_SO_4_	Anhydrite	Clinker		Aggreg.	SP
						0.85	2.5		
Plain	275	495	172	10	-	-	-	1375	9
2.5	276	428	173	10	10	69	69	1311	9
0.85	276	428	173	10	10	138	-	1311	9

**Table 4:** Designation and specifications of test members.

Designation	Clinker Particle Size	Curing Condition	Loading and Pre-Damage Status
V_28D-2.5	2.5 mm	28 days of air-drying	Loading up to ultimate state at 28 days
PLRL_28D-2.5		28 days of air-drying	Pre-loading up to 50% of ultimate load at 28 days + loading up to ultimate state at 28 days
RL_28 + 90D-2.5		28 days of air-drying followed by partial water curing for 90 days	Pre-loading up to 50% of ultimate load at 28 days + loading up to ultimate load 90 days after
V_28 + 90D-2.5		Air-drying until 28 + 90 days	Loading up to ultimate state after 28 + 90 days
V_28D-0.85	0.85 mm	28 days of air-drying	Loading up to ultimate state at 28 days
PLRL_28D-0.85		28 days of air-drying	Pre-loading up to 50% of ultimate load at 28 days + loading up to ultimate state at 28 days
RL_28 + 90D-0.85		28 days of air-drying followed by partial water curing for 90 days	Pre-loading up to 50% of ultimate load at 28 days + loading up to ultimate load 90 days after
V_28 + 90D-0.85		Air-drying until 28 + 90 days	Loading up to ultimate state after 28 + 90 days
P-V_28D	OPC	28 days of air-drying	Loading up to ultimate state at 28 days
P-PLRL_28D		28 days of air-drying	Pre-loading up to 50% of ultimate load at 28 days + loading up to ultimate state at 28 days
P-RL_28 + 90D		28 days of air-drying followed by partial water curing for 90 days	Pre-loading up to 50% of ultimate load at 28 days + loading up to ultimate load 90 days after
P-V_28 + 90D		Air-drying until 28 + 90 days	Loading up to ultimate state after 28 + 90 days

**Table 5:** Compressive strength and slump flow of considered mortars.

Series	Compressive Strength (σ)		Slump Flow (σ) (mm)
	7 Days (MPa)	28 Days (MPa)	
Plain	38.32 (1.12)	47.46 (1.48)	203 (8.2)
2.5	37.27 (0.89)	48.92 (1.59)	207 (8.0)
0.85	37.89 (1.03)	49.33 (1.56)	208 (8.1)

**Table 6:** Physical properties of concrete mixes.

Series	$f_{\mathrm{ck}}\;(\sigma )$		$E_{c}\;(\sigma )$		Slump (σ) (mm)	Air
	28 Days (MPa)	28 + 90 Days (MPa)	28 Days (MPa)	28 + 90 Days (MPa)		(%)
Plain	57.55 (2.01)	59.65 (1.98)	26,713 (1,219)	29,453 (1,410)	42.5 (2.10)	7.5
2.5	60.79 (2.08)	67.58 (2.11)	21,670 (1,002)	25,538 (987)	44.2 (2.06)	4.6
0.85	62.88 (2.14)	63.63 (2.18)	28,190 (1,145)	29,087 (1,328)	45.2 (1.97)	8.2

**Table 7:** Flexural test results of concrete mixes.

Series	Width of Failure Section (mm)		Height of Failure Section (mm)		Load (σ) (kN)		$f_{b}\;(\sigma )$ (MPa)	
	28 Days	28 + 90 Days	28 Days	28 + 90 Days	28 Days	28 + 90 Days	28 Days	28 + 90 Days
Plain	99.8	100.0	101.0	98.8	16.0 (0.92)	16.6 (1.01)	7.1 (0.31)	7.6 (0.18)
2.5	101.7	100.0	100.0	100.0	13.8 (0.73)	18.2 (1.12)	6.1 (0.27)	8.4 (0.31)
0.85	98.0	100.0	100.5	100.0	17.6 (0.77)	19.3 (1.09)	8.0 (0.29)	8.7 (0.28)

**Table 8:** Crack, yield and ultimate loads and failure patterns of test members.

Test Members	Crack Load (kN)	Yield (kN, mm)		Ultimate (kN, mm)		Ultimate/Yield		Failure Pattern
		Load	Displ.	Load	Displ.	Load	Displ.	
V_28D-2.5	5.63	242.18	7.93	267.90	18.10	1.11	2.28	Flexure
PLRL_28D-2.5	4.16	246.20	6.52	283.33	21.32	1.15	3.27	Flexure
RL_28 + 90D-2.5	11.54	238.97	8.39	288.00	38.58	1.21	4.60	Flexure
V_28 + 90D-2.5	9.96	245.02	7.48	281.01	26.02	1.15	3.48	Flexure
V_28D-0.85	2.78	234.94	7.93	277.88	20.02	1.18	2.52	Flexure
PLRL_28D-0.85	4.71	251.36	5.51	290.98	21.92	1.16	3.98	Flexure
RL_28 + 90D-0.85	11.79	233.91	5.81	291.90	41.94	1.25	7.22	Flexure
V_28 + 90D-0.85	9.42	245.59	7.28	289.55	37.02	1.18	5.09	Flexure
P-V_28D	6.17	240.44	8.52	268.29	20.23	1.12	2.37	Flexure
P-PLRL_28D	5.37	236.81	5.47	279.62	18.66	1.18	3.41	Flexure
P-RL_28 + 90D	11.32	237.08	6.94	282.60	41.78	1.19	6.02	Flexure
P-V_28 + 90D	5.92	239.54	8.26	278.75	33.72	1.16	4.08	Flexure

**Table 9:** Crack distribution density at bottom of specimens (/mm).

Test Member	2.5-Series	0.85-Series	P-Series
V_28D	0.0055	0.0065	0.0050
V_28 + 90D	0.0050	0.0075	0.0065
PLRL_28D	0.0075	0.0070	0.0065
RL_28 + 90D	0.0070	0.0060	0.0070

**Table 10:** Water flow test results.

Test Member	Initial Water Height (mm)	Final Water Height (mm)	Difference of Water Height (mm)
Healthy part	800	798	2
RL_28 + 90D-2.5		642	158
RL_28 + 90D-0.85		798	2
P-RL_28 + 90D		0	800
